# Combined MR direct thrombus imaging and non-contrast magnetic resonance venography reveal the evolution of deep vein thrombosis: a feasibility study

**DOI:** 10.1007/s00330-016-4555-4

**Published:** 2016-08-30

**Authors:** I. A. Mendichovszky, A. N. Priest, D. J. Bowden, S. Hunter, I. Joubert, S. Hilborne, M. J. Graves, T. Baglin, D. J. Lomas

**Affiliations:** 1 Department of Radiology, Addenbrooke’s Hospital, Cambridge, UK; 2 Department of Radiology, University of Cambridge, Cambridge, UK; 3 Department of Haematology, Addenbrooke’s Hospital, Cambridge, UK

**Keywords:** Magnetic resonance imaging, Venous thrombosis, Diagnosis, Longitudinal study, Clinical decision-making

## Abstract

**Objectives:**

Lower limb deep venous thrombosis (DVT) is a common condition with high morbidity and mortality. The aim of the study was to investigate the temporal evolution of the acute thrombus by magnetic resonance imaging (MRI) and its relationship to venous recanalization in patients with recurrent DVTs.

**Methods:**

Thirteen patients with newly diagnosed lower limb DVTs underwent MRI with non-contrast MR venography (NC-MRV) and MR direct thrombus imaging (MR-DTI), an inversion-recovery water-selective fast gradient-echo acquisition. Imaging was performed within 7 days of the acute thrombotic event, then at 3 and 6 months.

**Results:**

By 3 months from the thrombotic event a third of the thrombi had resolved and by 6 months about half of the cases had resolved on the basis of vein recanalisation using NC-MRV. On the initial MR-DTI acute thrombus was clearly depicted by hyperintense signal, while the remaining thrombi were predominantly low signal at 3 and 6 months. Some residual thrombi contained small and fragmented persisting hyperintense areas at 3 months, clearing almost completely by 6 months.

**Conclusions:**

Our study suggests that synergistic venous assessment with combined NC-MRV and MR-DTI is able to distinguish acute venous thrombosis from the established (old) or evolving DVT detected by ultrasound.

***Key Points*:**

• *MRI can distinguish between acute and evolving or chronic lower limb DVT*

• *Two advanced MRI techniques can follow the evolution of lower limb DVT*

• *MRI could be used to avoid an incorrect diagnosis of recurrent DVT*

• *MRI could help avoid the risks and complications of lifelong anticoagulation therapy*

## Introduction

Lower limb deep venous thrombosis (DVT) is a common condition with high morbidity and mortality encountered both in community and hospital care [1]. Despite current anticoagulation-based treatments, the recurrence rate after a first episode of DVT remains high, ranging between 20 % and 40 % [2–4]. Current management of a recurrent DVT is lifelong anticoagulation therapy, bringing with it both the additional cost and the burden of monitoring, as well as the well-recognized risks related to haemorrhage. These risks have to be balanced against the progression of a DVT with further complications such as pulmonary emboli that, in an important minority of cases, may be fatal [2, 4].

A clinically suspected DVT, often based on a positive D-dimer test and clinical scoring systems, is evaluated and followed up by compression ultrasound (CUS), now considered the standard diagnostic imaging test for lower limb DVT for the popliteal vein and above [5–8]. An acute symptomatic DVT is confirmed by CUS through the lack of compressibility of the common femoral, femoral and/or popliteal veins. However, the clinical presentation and diagnosis can be complicated by often ill-defined and occasionally absent symptoms that may accompany thrombus formation. In addition, many patients do not undergo follow-up imaging to confirm whether the affected vessels have fully or partially recanalised or remain occluded with chronic organized thrombus and increased collateral venous flow providing venous return.

Compression ultrasound has proven to be a simple, non-invasive, highly accurate, safe and cost-effective technique with high sensitivity and specificity of 89–100 % and 87–100 %, respectively, for DVT of the popliteal vein and above in symptomatic patients [4, 9]. Despite all its advantages, CUS cannot reliably determine the age of a thrombus and thus distinguish an acute recurrent DVT from a persisting previous thrombus in the same location. Interpretation of CUS findings is further complicated by the persistence of US abnormalities after the initial episode (80 % of patients after 3 months and 50 % of patients after 1 year from initial DVT diagnosis) [4, 7, 10, 11]. Quantitative thrombus measurements have been proposed as a way to distinguish between new and recurrent DVT that, although with poor interobserver agreement in initial studies, have improved in recent publications [12, 13]. However, limited information remains available on the temporal evolution of an acute thrombus and our ability to distinguish between a resolving (old) or recurring (new) DVT.

Magnetic resonance direct thrombus imaging (MR-DTI) has been proposed as an alternative that may help establish thrombus age and distinguish between an acute recurrent thrombus and a persisting (evolving) clot in the same location [14–19]. Several studies have shown that MR-DTI is a highly accurate diagnostic test for an acute symptomatic DVT [14, 15]. The technique is based on the paramagnetic properties of methaemoglobin, formed in a fresh thrombus, which decreases the longitudinal relaxation time (T1), resulting in high signal on T1-weighted images. The MR-DTI highlights this bright thrombus signal by nulling the signals from fat (using a water-selective excitation) and blood (due to its long T1). The technique has been shown to have a high sensitivity and specificity for proximal DVT of the lower limbs (sensitivity 97–100 %; specificity 100 %) and good reproducibility [18–20].

Non-contrast MR venography (NC-MRV) has also been shown to be effective in demonstrating the peripheral venous system and diagnosing DVT in the thigh through visualization of filling defects within the venous lumen [21]. However, this technique alone cannot reliably distinguish between an acute and an evolving thrombus, as both of these entities will appear as intraluminal filling defects.

The aim of this study was to evaluate the temporal changes that occur in T1-weighted signal intensity and venous morphology in patients with a first acute DVT using combined MR-DTI and NC-MRV at three different time points after diagnosis. The understanding of the temporal evolution, as well as the accurate diagnosis of an acute thrombus, is clinically important, as it may help distinguish between a new clot (formed at or adjacent to the site of a previous DVT) and an evolving (old) thrombus, directly informing the clinical decision of lifelong anticoagulation in patients with recurrent DVT.

## Materials and methods

### Patients

Thirteen patients (mean age 61 years, range 18–78 years; M/F = 11:2) with ultrasound-proven above-knee DVT (in the femoral vein and/or proximal popliteal vein) and no previous history of venous thrombosis or contraindications for MRI were recruited from the DVT service in our institution. The local ethics committee approved the study and all patients gave informed consent before undergoing MR imaging. The initial diagnosis of DVT was made on CUS by experienced sonographers as part of routine standard of care. Following patient recruitment, a member of the research team with ultrasound experience repeated the CUS study and the following parameters were recorded: the date/time of onset of symptoms, the patient’s (pro-thrombotic) risk factors, the length of thrombus and its relationship to the inguinal ligament and knee joint space. CUS was also performed at 3 months (visit 2) and 6 months (visit 3) on the same day as the MRI follow-up. On these visits, CUS was only used to establish the presence of the thrombus.

MR imaging was performed on a 1.5-T scanner (Discovery MR450, GE Healthcare, Waukesha, WI) using a 12-channel phased array coil. The first examination (visit 1) was within 7 days of diagnosis, and further examinations were performed after 3 months (visit 2) and 6 months (visit 3). During this 6-month period all the patients received standard anticoagulant therapy (warfarin) in accordance with local protocols.

### MR examination

For each examination, NC-MRV was acquired using Acceleration-Dependent Vascular Anatomy for Non-Contrast-Enhanced MR Venography (ADVANCE-MRV) [22, 23]. This technique uses subtraction of velocity- and acceleration-dependent angiography methods, as described previously [24, 25]. Dark-vein images are subtracted from bright-vein images to obtain vein-only images. The bright-vein images were acquired using an acceleration-sensitized acquisition to suppress the arterial blood, without suppressing venous blood [22, 23]. The dark-vein (velocity-sensitized) images were acquired using two consecutive velocity-sensitization modules with differing effective first gradient moments [22, 23]. To further ensure good venous suppression, four of these dark-vein image volumes were automatically acquired together and combined to give homogeneous venous signal [22, 23]. The effective first gradient moments used to generate the dark-vein image volumes were 1.2, 0.6, 0.3 and 0.15 μTs^2^/m. Additionally, a bright-artery image volume was acquired and used to generate an artery-only image volume by automated subtraction of the dark-artery (bright vein) images described above.

For these acquisitions, the image readout was a 3D balanced-SSFP (oblique coronal orientation, flip angle 65°, echo time (TE) 1.7 ms, repetition time (TR) 3.7 ms, acquisition matrix 288 × 288 × 20, reconstruction matrix 512 × 512 × 40, field of view (FoV) 40 × 40 cm, slice thickness 2.4 mm). The acquisition was cardiac gated using peripheral-pulse triggering with a trigger delay set to peak arterial flow. Parallel imaging (ASSET) was used with an acceleration factor of 2. The acquisition time was 40 heartbeats per volume, or 240 heartbeats for one bright-vein, four dark-vein and one bright-artery image volumes. To avoid flow and heterogeneity-related artefacts near the edges of the field of view, two separate acquisitions covering the upper and lower halves of the thigh were performed, with a combined acquisition time of 480 heartbeats (8 min at 60 bpm). Based on the above matrix sizes, the acquired and reconstructed voxel dimensions were 1.38 × 1.38 × 2.4 mm^3^ and 0.78 × 0.78 × 1.2 mm^3^ respectively.

MR-DTI was performed using a 3D inversion-prepared fast gradient-echo acquisition with a water-selective excitation (1-2-1 binomial pulse sequence). The image volumes were acquired in coronal orientation and the image parameters were optimized by numerical simulation to ensure high signal from short-T1 thrombus while suppressing the signal from long-T1 blood [26]. The acquisition used centric k-space ordering, acquiring half the slice-encodes from each read-slice k-space plane per shot. The acquisition parameters were inversion time (TI) 340 ms, TE 6.3 ms, TR 12.2 ms, flip angle 25°, centric ordering, acquisition matrix 320 × 288 × 96, FoV 40 × 40 cm^2^, slice thickness 2 mm, 320 ms delay after each shot. Parallel imaging (ASSET) was used with an acceleration factor of 2. Zero-filling interpolation was used in-plane to give a reconstructed volume size of 512 × 512 × 96. The acquisition time was 5 min 59 s.

### MR image analysis

The MR-DTI volumes were reformatted to match the NC-MRV images. An experienced radiologist (DJL) assessed the NC-MRV images using an Osirix workstation (Pixmeo, Berne, CH) to determine the location and length of the venous occlusion (complete or partial, above-knee only). This assessment was performed principally using the subtracted NC-MRV images; however, the arterial images, as well as the unsubtracted bright- and dark-vein images, were also available to aid the diagnosis where needed.

Subsequently, an assessment of the location and length of any high-signal regions on the matching MR-DTI images was performed and these were compared with the NC-MRV images to evaluate to what extent thrombus length and location matched on both techniques. The individual-slice images were evaluated with reference to curved-reformat images, as required where the thrombi curved significantly out of the image plane. Any mismatch in thrombus extent or location was recorded. All discontinuities in high signal T1 regions were noted and the tract lengths were summed over the affected region on the matching NC-MRV.

## Results

**Fig. 1:**
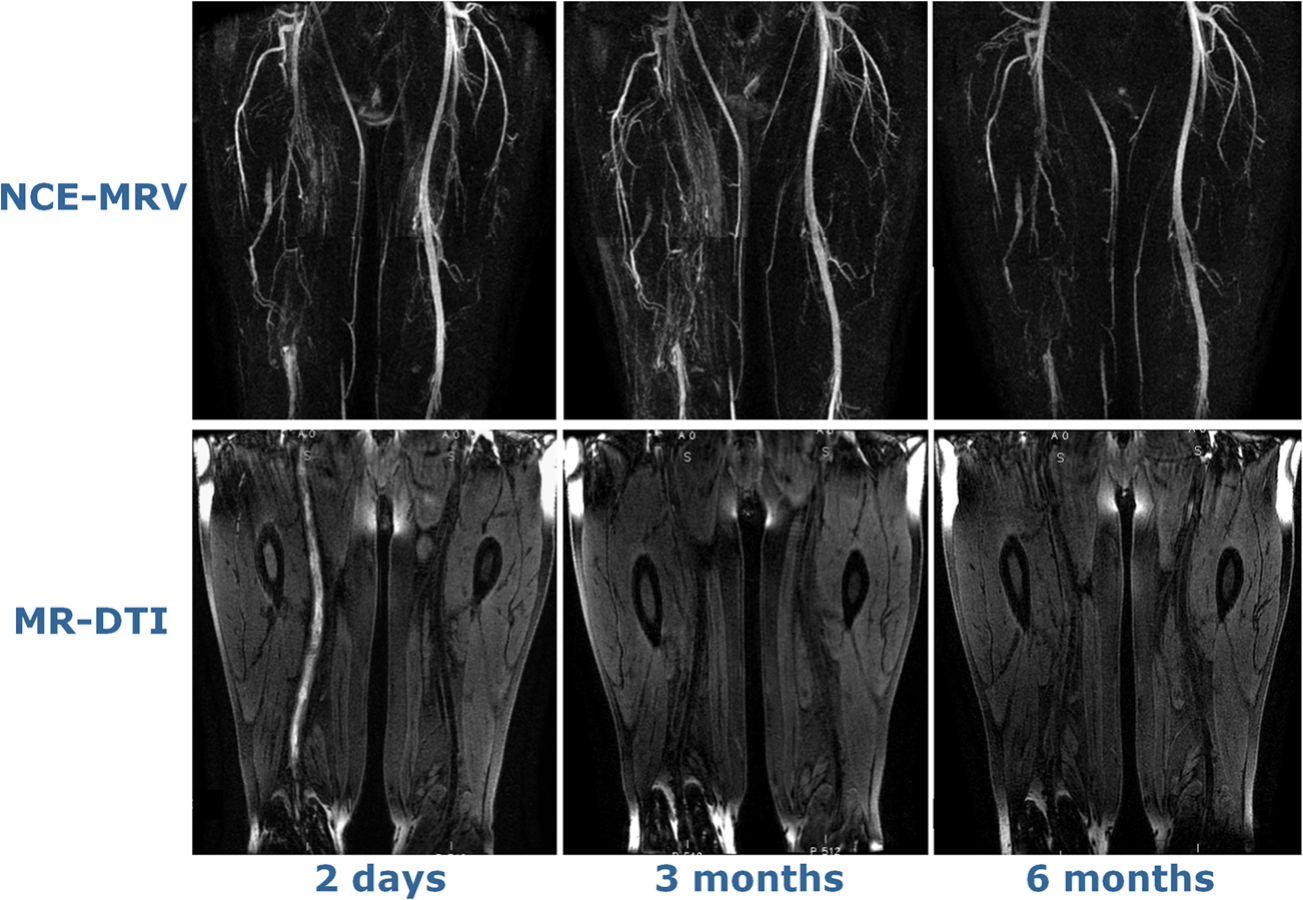
Images for an example patient showing the evolution of the thrombus signal. The NC-MRV image (a composite MIP from the two table positions) shows an occluded right femoral vein at all three time-points, but the thrombus is visible on MR-DTI only for the first examination. MR-DTI images are shown as curved-plane reformats

The femoral vein occlusion by thrombus was identified in all cases on NC-MRV and matched the ultrasound findings for location and extent. The thrombus length was between 6.4 and 40.0 cm (mean 27.7 cm) on the first visit. On visit 2, thrombus was no longer present in 4/13 cases, had reduced in length in a further 6/13 cases (range 3.8–30.3 cm, mean 15.9 cm) and was unchanged in the remaining three cases (range 32.4–39.0 cm). On visit 3, only 6/13 cases still had thrombus present (range 17.9–39.0 cm, mean 30.4 cm). Figures 1 (patient G) and 2 (patient C) show NC-MRV and MR-DTI images in two patients with venous occlusion on all three visits, while Fig. 3 shows the measured lengths of the occluded region on the NC-MRV and the corresponding MR-DTI images for each visit and each patient. All thrombus length measurements are summarized in Table 1.

**Fig. 2:**
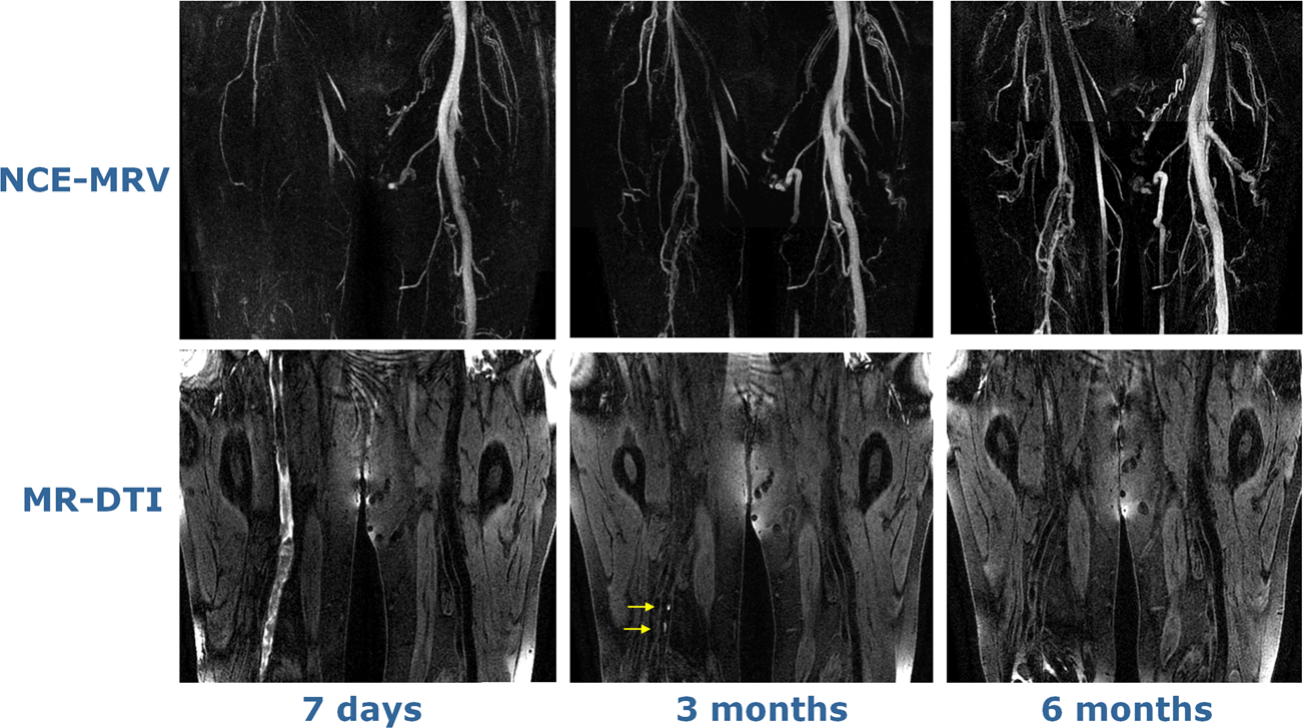
MIP images of the NC-MRV showing an occluded right femoral vein at all three visits; curved-plane reformats of the MR-DTI show the whole thrombus at visit 1, but only tiny fragments at visit 2 (*arrows*)

**Fig. 3:**
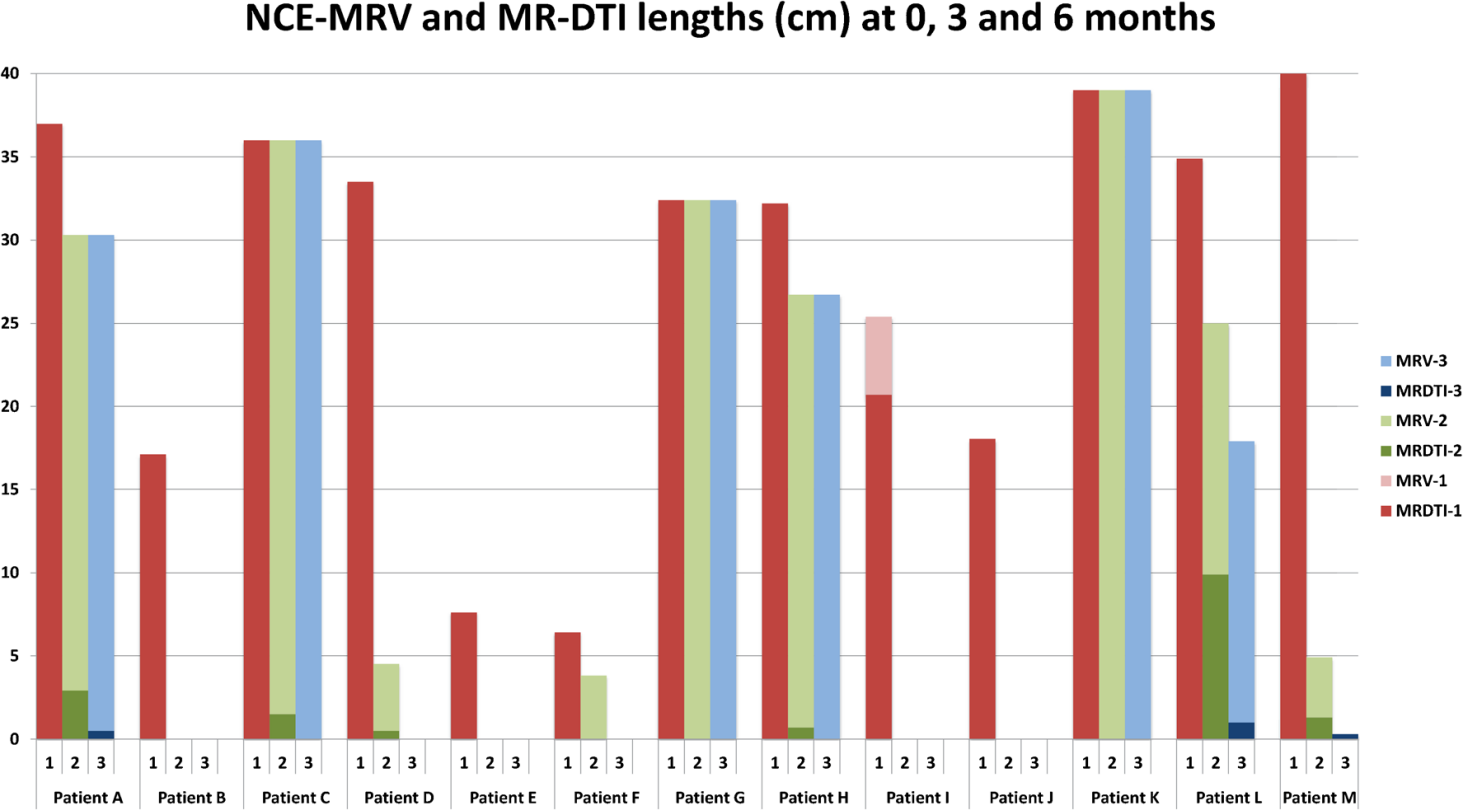
Measured lengths on NC-MRV and MR-DTI images. The darker colours for each patient and visit correspond to the measurements performed on MR-DTI images (*bottom bars*) while the NC-MRV measurements are illustrated in lighter colours (*upper bars*). If, in the same visit, the MR-DTI and NC-MRV measurements were identical, the MR-DTI value only was presented. If only NC-MRV values are shown then no measurable segment was seen on MR-DTI images

**Table 1:** NC-MRV (MRV-) and MR-DTI (MRDTI-) lengths (cm) in all patients and visits

Visit	Visit 1 (0 months)		Visit 2 (3 months)		Visit 3 (6 months)	
Patient	MRV-1	MRDTI-1	MRV-2	MRDTI-2	MRV-3	MRDTI-3
Patient A	37	37	30.3	2.9	30.3	0.5
Patient B	17.1	17.1	0	0	0	0
Patient C	36	36	36	1.5	36	0
Patient D	33.5	33.5	4.5	0.5	0	0
Patient E	7.6	7.6	0	0	0	0
Patient F	6.4	6.4	3.8	0	0	0
Patient G	32.4	32.4	32.4	0	32.4	0
Patient H	32.2	32.2	26.7	0.7	26.7	0
Patient I	25.4	20.7	0	0	0	0
Patient J	18.04	18.04	0	0	0	0
Patient K	39	39	39	0	39	0
Patient L	34.9	34.9	25	9.9	17.9	1
Patient M	40	40	4.9	1.3	0	0.3

**Fig. 4:**
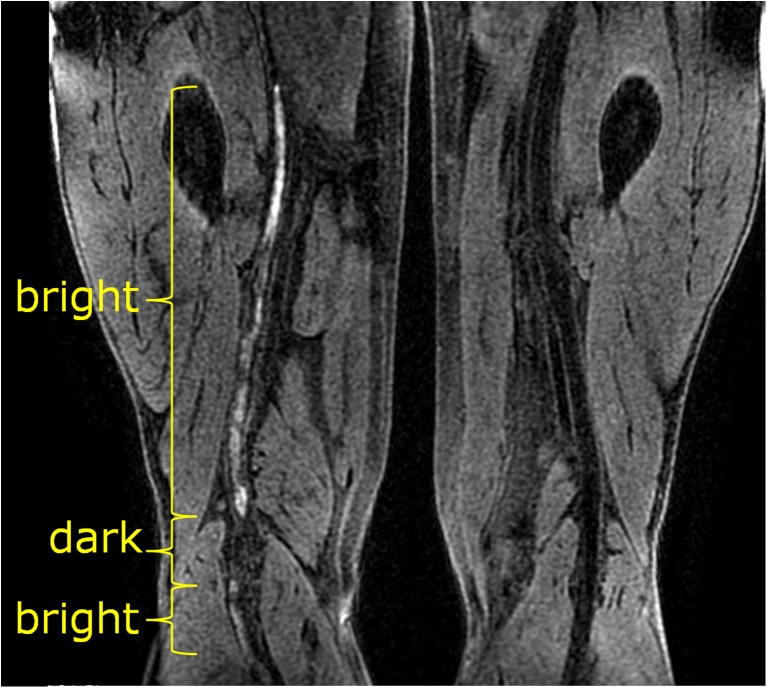
MR-DTI (curved-plane reformat) of the only case where the bright-signal region on examination 1 did not fully match the occlusion observed on NC-MRV. The thrombus contained a dark-signal region near the knee with bright-signal regions both proximal and distal to it. This central dark-signal region may contain older thrombus

**Fig. 5:**
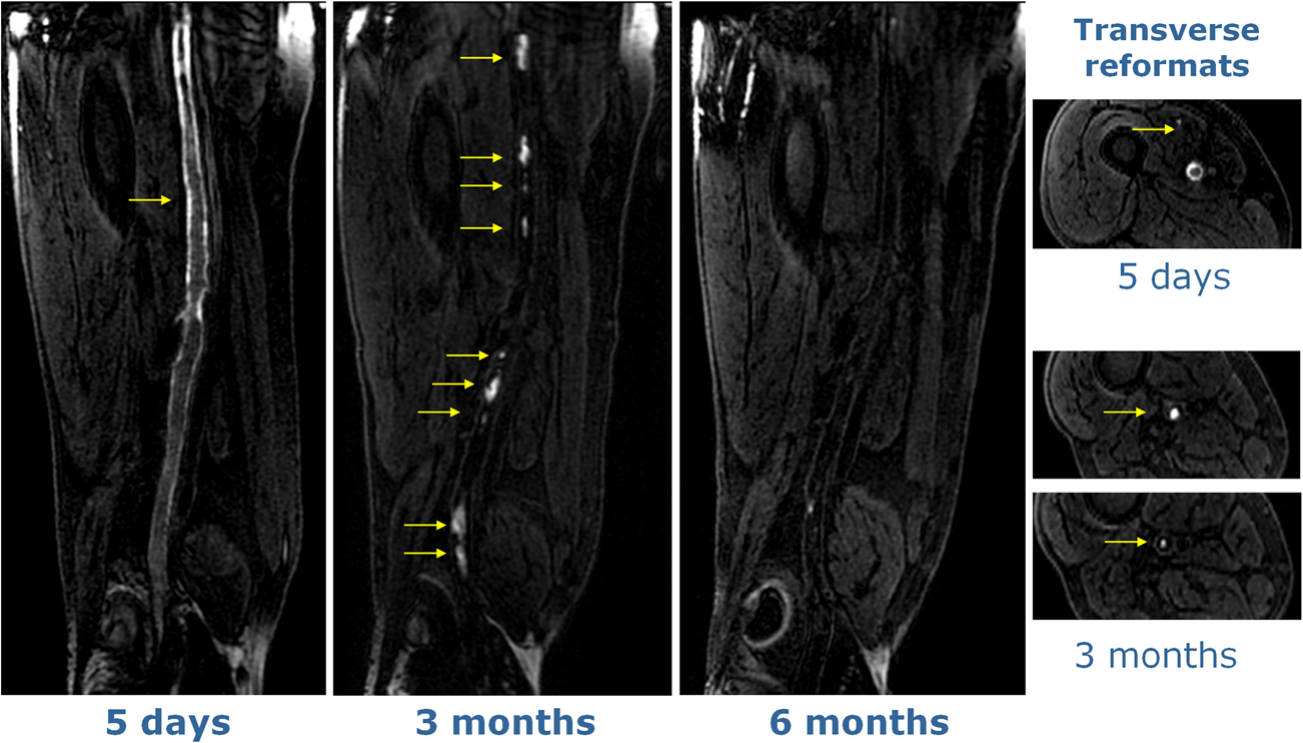
MR-DTI images at all three time-points for the case showing bright-signal fragments at visit 2 (3 months). Transverse reformats demonstrate that these fragments are located in the centre of the thrombus, suggesting that they represent evolution of the existing thrombus rather than development of new thrombus. In contrast, the hyperintense signal for the early time-point (visit 1) occurs around the edge of the thrombus

On the first visit, high signal at MR-DTI was found to match spatially the NC-MRV-defined occlusion in all but one case. This exception case (patient I) is shown in Fig. 4 and is discussed below. On the subsequent second and third visits, the thrombus was either not visualized or only small/fragmented bright signal areas were seen on MR-DTI—less than 29 % (visit 2) and 3 % (visit 3) of the original thrombus length. The case with most residual bright thrombus signal on visits 2 and 3 is shown in Fig. 5 (patient L). Recanalisation, as assessed on the NC-MRV images, occurred in seven patients, but in six patients the superficial femoral vein remained partially or completely occluded.

## Discussion

Our study is in line with previous work and confirms the potential value of MR-DTI in the diagnosis and follow-up of acute DVT involving the femoral and proximal popliteal veins by detecting the presence and changes in methaemoglobin content of the recently formed thrombus [18]. It demonstrates that by 3 months from the thrombotic event about a third of the thrombi had resolved and by 6 months only about half of the cases had residual thrombus present. When following the evolution of methaemoglobin changes over time, at 3 months less than half of the patients showed persistent, albeit small and fragmented, hyperintense areas at the site of the thrombus that almost completely cleared at the 6-month MR-DTI examination, consistent with the expected evolution of methaemoglobin.

The length and location of the thrombus matched in all patients on the NC-MRV and MR-DTI images on the first visit, except in one case (patient I shown in Fig. 4). In this patient, the proximal and distal ends of the thrombus were clearly depicted on both the NC-MRV and MR-DTI images, but the MR-DTI signal was discontinuous distally in the region of the knee. However, the extent of thrombus could still be reliably measured, as an absence of flow was demonstrated in the region of MR-DTI discontinuity.

Figure 5 (patient L) shows persistent occlusion of the vein on all 3 visits with multiple short discontinuous hyperintense segments remaining at 3 months (visit 2) and reducing substantially in size at 6 months (visit 3). At visit 2, this exam contains the most high signal MR-DTI fragments of any examination in this study. These fragments are discontinuous and are more likely to represent residual fragmented thrombi from the initial thrombotic event, as the vein remained completely occluded (confirmed by the NC-MRV) and the bright foci were located more centrally in the vein as opposed to the peripheral “ring-like” distribution of the bright signal on the surface of the thrombus at visit 1.

Three patients (A, L and M) had high signal intensity foci on MR-DTI images both at 3 and 6 months. In contrast to the other two patients (A and L) where these hyperintense fragments were accompanied by occlusion on NC-MRV, patient M showed no occlusion on the corresponding NC-MRV images. In this latter case (patient M), the bright fragments could represent either “residual” thrombus from the initial DVT or a “new” thrombus (perhaps related to an area of endothelial damage).

Our work has several strengths. We designed a longitudinal study to characterize the evolution of acute “de novo” presentations of deep vein thrombosis with two non-invasive non-contrast MR methods: a vascular technique (NC-MRV) and a thrombus-characterization technique (MR-DTI). Despite being challenging to implement because of the complexities of arterial and venous haemodynamics, we were able to perform these techniques within the constraints of a clinical workflow and acquire diagnostic quality NC-MRV and MR-DTI in a clinically acceptable examination time. We successfully confirmed the expected evolution of lower-limb DVT in the majority of patients. These findings, if confirmed in a larger cohort study, could improve the diagnosis of recurrent DVT that is typically treated with lifelong anticoagulation.

The limitations of this study are the small number of patients and the use of compression US alone as the “gold standard” for thrombus detection (as invasive venogram studies are no longer performed for DVT diagnosis in our clinical practice). The recruitment criteria were designed to avoid patients with prior DVT but it is possible that an asymptomatic DVT may have occurred previously in our population as discussed regarding patient I.

Future work will concentrate on further optimization of this technique, expanding the number of patients and investigating the relationships between the observed small MR-DTI hyperintensities and the clinical evolution and risk profile of these patients.

MR-DTI, in combination with other methods such as NC-MRV or US, may in future prove to be a valuable tool for distinguishing between acute and chronic thrombus in cases of suspected recurrence, potentially improving clinical management.

## Conclusion

This initial study demonstrates that, in patients with a lower limb DVT, consistency between MR-DTI and NC-MRV evaluations of thrombus size and location suggests that the thrombus is acute, while a large discrepancy, or absence of high signal on MR-DTI for NC-MRV-observed thrombi, suggests an older, pre-existing thrombus. These non-invasive MRI techniques used in patients with symptoms of lower limb DVT might help in future avoid an incorrect diagnosis of recurrent DVT thereby avoiding the risks and complications associated with lifelong anticoagulation therapy.

## Abbreviations

ADVANCE-MRV: Acceleration-dependent vascular anatomy for non-contrast-enhanced MR venography
CUS: Compression ultrasonography
DVT: Deep venous thrombosis
MR-DTI: Magnetic resonance direct thrombus imaging
NC-MRV: Non-contrast MR venography
